# The effects of propolis extract on ovarian tissue and oxidative stress in rats with maternal separation stress 

**Published:** 2017-08

**Authors:** Atefeh Arabameri, Hamidreza Sameni, Ahmadreza Bandegi

**Affiliations:** 1 *Department of Biology, Faculty of Basic Sciences, Islamic Azad University, Damghan, Iran.*; 2 *Nervous System Stem Cells Research Center, Department of Anatomical Sciences, Faculty of Medicine, Semnan University of Medical Sciences, Semnan, Iran.*; 3 *Nervous System Stem Cells Research Center, Department of Biochemistry, Faculty of Medicine, Semnan University of Medical Sciences, Semnan, Iran.*

**Keywords:** Propolis, Stress, Ovary, Corticosterone, Oxidative stress, Rats

## Abstract

**Background:**

Stress in infancy has dramatic effects on different systems, including the nervous system, endocrine, immune, reproductive and etc.

**Objective:**

The purpose of this study was to investigate the effects of extract of Iranian propolis (EIP) on ovarian tissue and oxidative stress in rats with maternal separation stress.

**Materials and Methods:**

48 immature female rats were divided randomly into six groups. 1) Control group, 2) Control group+saline, 3) Stress group, includes infants that were separated from their mothers 6 hr/day, the 4^th^, 5^th^ and 6^th^ groups consisted of infants who in addition to daily stress received 50, 100 and 200 mg/kg of EIP, respectively. Then serum corticosterone, 17-beta-estradiol, malondialdehyde, total superoxide dismutase, glutathione peroxidase and ferric reducing antioxidant power levels were measured. The ovarian sections were stained by H&E, PAS, and TUNEL methods and were studied with optical microscopy.

**Results:**

Stress increased the blood serum corticosterone levels and 17-beta-estradiol reduced significantly (p<0.001) and EIP prevented from this changes (p<0.01). EIP significantly increased the number of ovarian follicles, oocytes and oocytes diameter in neonatal rat following stress (p<0.01). EIP also significantly decreased the number of atretic follicles, TUNEL+granulosa cells, malondialdehyde levels and increased ferric reducing antioxidant power, total superoxide dismutase and glutathione peroxidase serum levels in neonatal rats following stress. The dose of 200 mg/kg EIP was more effective.

**Conclusion:**

This Study showed that the Iranian Propolis significantly could prevent oxidative stress and histopathological changes in the ovary of the neonatal rat the following stress.

## Introduction

Stress is any threatening event that causes to reveal the behavioral and physiological responses in an individual (1). The stresses during pregnancy and after the birth are related to many mental, behavioral and cognitive abnormalities in humans and animals (2). The types of stress include, physical (motion limitation, foot shock, exercise, cold, intense light, etc.), metabolic, immunological, cardiovascular and mental stress and so on can be pointed (3). It has been reported that female reproductive system is very sensitive to stress and effect of stress on gonads in adults may be reversible but it may not be true during initial stages of development. Thus, stress experienced during neonatal or pre-pubertal phases might have serious side effects on the ovarian follicular development (4). In the stressed conditions, two systems are active in the body: one is a sympatho-adreno-medullary system that releases catecholamine's (epinephrine and norepinephrine), and the other is hypothalamus-pituitary-adrenal axis (HPA) that releases glucocorticoids (corticosterone in rodents and cortisol in humans) by stimulating the adrenal cortex (5-8).

Glucocorticoid receptors are found in hypothalamic gonadotropin-releasing hormone (GnRH) neurons and cells of pituitary gonadotropin. Glucocorticoids with the same concentration as stress state block the level of GnRH in the pituitary gland and subsequently, the responsiveness of gonadotropins to GnRH is also reduced, thus the reduction of LH level and consequently the lack of ovulation and the defect of the menstrual cycle happen (9). Glucocorticoids also cause to reduce estrogen secretion by blocking aromatase activity in granulosa cells influencing not only steroid-making but also reduces the expression of estrogen and progesterone receptors and may cause to damage the ovary and leads to disruption or delay in pregnancy (9).

There exists a reciprocal relationship between HPA and the hypothalamic-pituitary-gonadal axes. For instance, both testosterone and oestrogen modulate the response of the HPA axis, while activation of the stress axis, especially activation that is repeating or chronic, has an inhibitory effect upon oestrogen and testosterone secretion. Alterations in maternal care can produce significant effects on both hypothalamic-pituitary-gonadal and HPA physiology and behavior in the offspring at adulthood (6).

Stress, excessive secretion of glucocorticoids can cause cellular oxidative stress (10). In the state of oxidative stress, high and abnormal level of Reactive oxygen species (ROS), such as free radicals (hydroxyl, nitric oxide, superoxide) or non-radicals (H_2_O_2_ and lipid peroxide), can damage to the certain molecules and the cell components such as lipids, nucleic acids and proteins, leading to the cell death as necrosis and apoptosis (10, 11). After lipid peroxidation, intermediate compounds that are the main characteristic of the oxidative stress process, have been created in the cell; Malondialdehyde (MDA) can be referred as one of these compounds. Naturally, in the cells, there are the enzymes such as Superoxide Dismutase, Catalase, Glutathione peroxidase and Glutathione Reductase that are responsible for antioxidant defense (11). Reports have shown that the oxidative stress have the important negative effects on female fertilities and health of gametes and the pharmacological or nutritional interventions as an effective strategy protecting female fertilities from the negative effects of ROS and oxidative stress (9).

Propolis is a red or brown resinous substance that honey bee’s collect from the tree buds, leaves, sop flows and other botanical sources and then the bees add wax and their other secretions to it. It is used as a sealant for unwanted open spaces in the hive and is used as a traditional herbal medicine in many countries. Many studies confirmed the biological and pharmacological activities including its anti-cancer, anti-inflammatory, antibacterial and antiviral effects, anti-Parkinson’s disease and antioxidant properties and attributed these characteristics to its various chemical compounds (10, 12-15).

Our earlier studies have shown that the Iranian propolis extract could enhance the antioxidant levels and histopathological changes in the kidneys and control blood glucose and modulate some of the biochemical factors of diabetic rats, also prevent from increasing serum corticosterone and brain MDA levels and from decreasing ferric reducing antioxidant power (FRAP), superoxide dismutase (SOD) and glutathione peroxidase (GPx) levels in brain under prenatal stress (16-18). Propolis is one of the strongest natural antioxidants. The measured antioxidant activity of propolis extract in units of oxygen radical absorbance capacity is four times greater than vitamin E (19). These effects are mainly due to of high concentrations of polyphenols and flavonoids that are the most important active medicinal and antioxidant compounds in propolis. Since many disorders during puberty and adulthood are caused by the various stresses which occur in prenatal and infancy periods. Therefore, finding the compounds that can control the harmful effects of the stress on the different organs, including the ovaries has the extremely high importance. 

Regarding the crucial and decisive role of the ovary in the fertility and the powerful antioxidant properties of propolis, the aim of this study was to investigate the protective effects of hydro-alcoholic extract of Iranian propolis (EIP) on ovarian tissue and oxidative stress in rats with maternal separation stress. 

## Materials and methods

**Animals and experimental design**

All procedures were carried out in the laboratory of biochemistry and stem cells research center of Semnan University of Medical Sciences, Semnan, Iran. A total of 48 female Wistar rats at the age of 15 days and the approximate weight of 20±5 were obtained from the laboratory animal center of Semnan University of Medical Sciences. The rats were maintained under constant conditions: light period (12 hr light/dark), relative humidity of 45-55%, controlled temperature of 22±2^o^C and free enough access to standard diet and water in plastic cages.

In this experimental study, the animals were randomly divided into six groups (n=8/each) as follows: 1) Control group (C) included 21-day pups without any intervention, 2) Sham group included 15-day pups that were beside their mothers during this period and received 0.1 ml saline solution daily, 3) Stress group (S), included 15-day pups that were separated from their mothers 6 hr/day during this period, 4) the S+P50 group which were under stress and received 50 mg/kg EIP daily, 5) the S+P100 group which were under stress and received 100 mg/kg EIP daily, and 6) S+P200 group which were under stress and received 200 mg/kg EIP daily (16-18).

The animals in groups C and Sham didn't have any stress and had enough diet and water, and the animals in the third to sixth groups were separated from their mothers 6 hr/day, were kept in separate cages with suitable beds and their injections were done intraperitoneally daily. The timing of separation from the mothers (6 hr/day) was randomly changed every day to avoid habituation (for example, as the periods from 8AM-14PM, 9AM-15PM, and so forth). The EIP was injected to the animals of the fourth, fifth and sixth groups intraperitoneally for seven days (from 15^th^ day to 21^st^ day after birth) before stress. The right ovaries were fixed in 4% paraformaldehyde, embedded in paraffin. They were then serially sectioned (5 µm), mounted on glass slides for histological and immunohistochemical studies. The blood sample was collected, serum was separated, stored at -20^o^C until corticosterone, and 17-beta-Estradiol concentration was determined (20, 21).

**Preparation of propolis extract**

In this investigation, propolis was collected from the bee-hives located in different parts of the Semnan province and verified by the agricultural organization. Extracts were prepared according to the method of Greenaway (23). In summary, the major components of propolis were chopped into small pieces, mixed (25 gr) with 250 ml of 80% ethanol, and incubated at room temperature for 48 hr with shaking (150 rpm). 

The extract was clarified twice by Whatman grade 42 filter paper and ethanol was evaporated using a rotary vacuum evaporator to obtain the purified propolis extract. The EIP concentrations were measured and diluted to the required dilutions (weight to volume) using 10% ethanol. The extracts were stored at 2-8^o^C under protective light conditions and warmed to room temperature just before injecting (22, 23). 

**Assay of serum concentration corticosterone and 17-beta-estradiol**

The corticosterone and 17-beta-estradiol serum concentration were determined by enzyme linked immunosorbent assay (ELISA) using the kits Corticosterone, ELISA, DRG, Marburg, Germany and 17-beta estradiol, ELISA, Bolden, England. The sensitivity of these kits is 0.39 nanograms per milliliter. To do this, the blood was collected from the hearts of the animals under complete anesthesia 24 hr after the last injection at the dose of 80/20 mg/kg Ketamine/Xylazine and after centrifugation at 300 rpm for 20 min; the obtained serum was stored at -20^o^C until measuring the hormone levels (24). 

**Assay of antioxidant activity**

After EIP treatment, all the pups were anesthetized by administering ketamine/ xylazine (80/20 mg/kg) intraperitoneally. After opening the abdominal cavity, the left ovary was removed, washed with physiological saline and was homogenized (10% w/v) in cold saline (1.15 M KCl) to prepare for the assay for activity of antioxidant enzymes. The homogenates were centrifuged at 20,000× gr for 10 min at 4^o^C. The supernatants were collected and used for assessment of Ferric reducing antioxidant power, MDA levels, T-SOD (Total superoxide dismutase), and GPx, activities (Randox Laboratories, Shanghai, china) (25-26). 

**Histological evaluation**

To study the Histomorphometry and to count of oocytes and ovarian follicles, the ovaries were removed from the body and their weights were determined after blood collection. The right ovaries fixed in 4% paraformaldehyde, were processed according to the standard histological method and 5μm thick serial paraffin sections were cut and stained with hematoxylin and eosin. Different categories of ovarian follicles were counted according to Pedersen and Peters methods (27). Briefly, every fifth ovary section was scanned under a dot side-digital virtual microscope, and the number of follicles in the entire section was counted. To avoid multiple counts of the same follicle, only those with a visible oocyte nucleus were included. 

In this manner, in a slice of the ovary, the primitive follicles were counted from each 4 slices and the unilamellar primary follicles of every 6 slices. But counting multilamellar primary follicles, pre-antral and antral follicles is in this way that the follicles which contain only oocytes with the perfect size in each slice of the ovary are counted and so the repeated counting of mentioned follicles can be avoided in other slices (28, 29). Atretic follicles were also counted and identified according to Greenwald's described morphological method using hematoxylin-eosin staining, serial sections and allocated terminal deoxynucleotidyl transferase dUTP nick end labeling (TUNEL) method (30, 31).

**TUNEL assay**

The follicular atresia, determined by the presence of more than 5% pycnotic granulosa cells (apoptotic cells), was further characterized by conducting TUNEL kit (In situ cell death detection kit, POD, Roche Germany) was used in order to identify the apoptotic cells. In this way, to determine the cell apoptosis, TdT enzyme is able to label 3ـ׳ OHs which have achieved the free form by breaking down the DNA during apoptosis. These sites were labeled with conjugated dUTP by fluorescein, and then the sites containing fluorescein were detected by anti-fluorescein antibody connected to POD. 

If there is a breakage in the DNA and the fluorescein is connected, fluorescein antibody is attached to these sites and POD in this antibody can make the brown color in reaction with the di-amino benzidine substrate. The cores of apoptotic cells become brown in this method. The number of TUNEL positive and negative granulosa cells were counted in randomly selected areas in each section and expressed in terms of percentage of TUNEL positive and negative cells (32).

**Ethical consideration**

All experimental protocols were approved by the ethics committee of Semnan University of Medical Sciences, Semnan, Iran (IR.SEMUMS.REC.1392.126), 

**Statistical analysis**

All data were expressed as mean±SEM. The quantitative data have been analyzed using the SPSS software (Statistical Package for Social Sciences, version 20.0, SPSS Inc, Chicago, Illinois, USA). Data from experiments with more than two independent variables have been analyzed using the analysis of variance (ANOVA) followed by the Tukey–Kramer post-hoc tests. The significant difference was considered between the groups at a significance level of p<0.05.

## Results

**Effect of postnatal stress and EIP on body weight**

**Figure 1:**
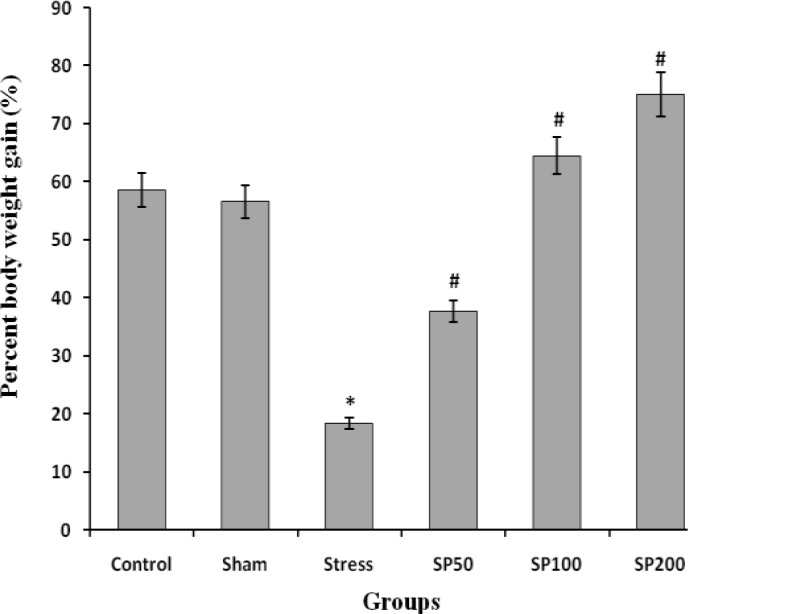
Effects of maternal separation stress and Iranian propolis extract on body weight in the rat neonates. Control group (Control), control group+saline infusion (Sham), stress group + saline infusion (stress), stress group+50 mg/kg propolis extract (SP50), stress group+100 mg/kg propolis extract (SP100) and stress group+200 mg/kg propolis extract (SP200). * P<0.001, compared to the Control and Sham groups, # P<0.001compared to the Stress group. All data are presented as Mean ± SEM, n=8

The mean body weight and percent body weight gain in S (stress) group were significantly lower than the control groups. Treatment with EIP at doses of 50, 100 and 200 mg/kg were significantly increased body weight (F=6.428, p=0.001, Figure 1). 

**Effect of postnatal stress and EIP on serum corticosterone and 17-beta-estradiol levels**

**Figure 2:**
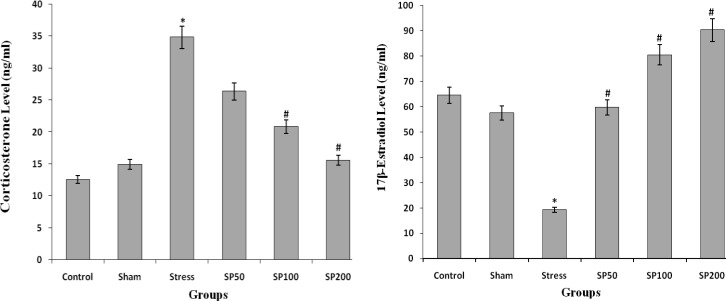
Effects of maternal separation stress and Iranian propolis extract on corticosterone level (ng/mL) and 17 beta-estradiol level (ng/mL) in the rat neonates. Control group (Control), control group + saline infusion (Sham), stress group+saline infusion (Stress), stress group+50 mg/kg propolis extract (SP50), stress group+100 mg/kg propolis extract (SP100) and stress group+200 mg/kg propolis extract (SP200). * P<0.001, compared to the Control and Sham groups, # P<0.001, compared to the Stress group. All data are presented as Mean ± SEM, n=8

The mean serum concentration of corticosterone was significantly higher in stressed rats compared to healthy and control groups. Serum corticosterone levels significantly reduced in the neonates that had stress and received EIP at doses of 100 and 200 mg/kg (F=6.211, p=0.001, Figure 2). Stress also reduced the amount of 17-beta-estradiol in the blood serum of the neonates in comparison with the sham and control groups. Serum 17-beta-estradiol levels significantly increased in the neonates that had stress and received EIP at doses of 100 and 200 mg/kg (F=6.729, p=0.01, Figure 2).

**Effect of postnatal stress and EIP on follicular and oocyte development**

The mean number of primordial, primary and secondary follicles in the stressed rats was significantly lower than others groups. The treatment of stressed pups with EIP (100 & 200 mg/kg) significantly increased the number of different types of the follicles (F=4.235, p=003; F=9.527, p=001; F=9.097, p=001 respectivly; Table I). The mean number and diameter of oocytes were decreased significantly in stressed rats in comparison with the others groups. The treatment of stressed pups with EIP (100 and 200 mg/kg) significantly increased the number and diameter of the oocytes (F=2.627, p=0.032; F=3.100, p=0.016 respectively; Table I).

**Effect of postnatal stress and EIP on apoptosis of granulosa cells and atretic follicles**

**Figure 3:**
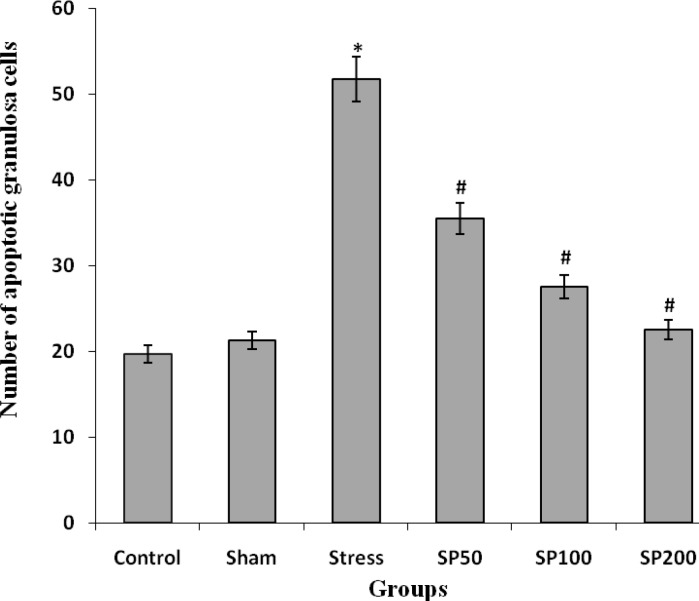
Effect of propolis on the number of TUNEL positive granulosa cells (in 0.022 mm2 of the ovary surface) in the ovaries of a rat with neonatal stress. Control group (Control), control group+saline infusion (Sham), stress group+saline infusion (Stress), stress group+50 mg/kg propolis extract (SP50), stress group+100 mg/kg propolis extract (SP100) and stress group+200 mg/kg propolis extract (SP200). * P<0.01, compared to the C and Sham groups, # P<0.001, compared to the S group. All data are presented as Mean±SEM, n=6

**Figure 4:**
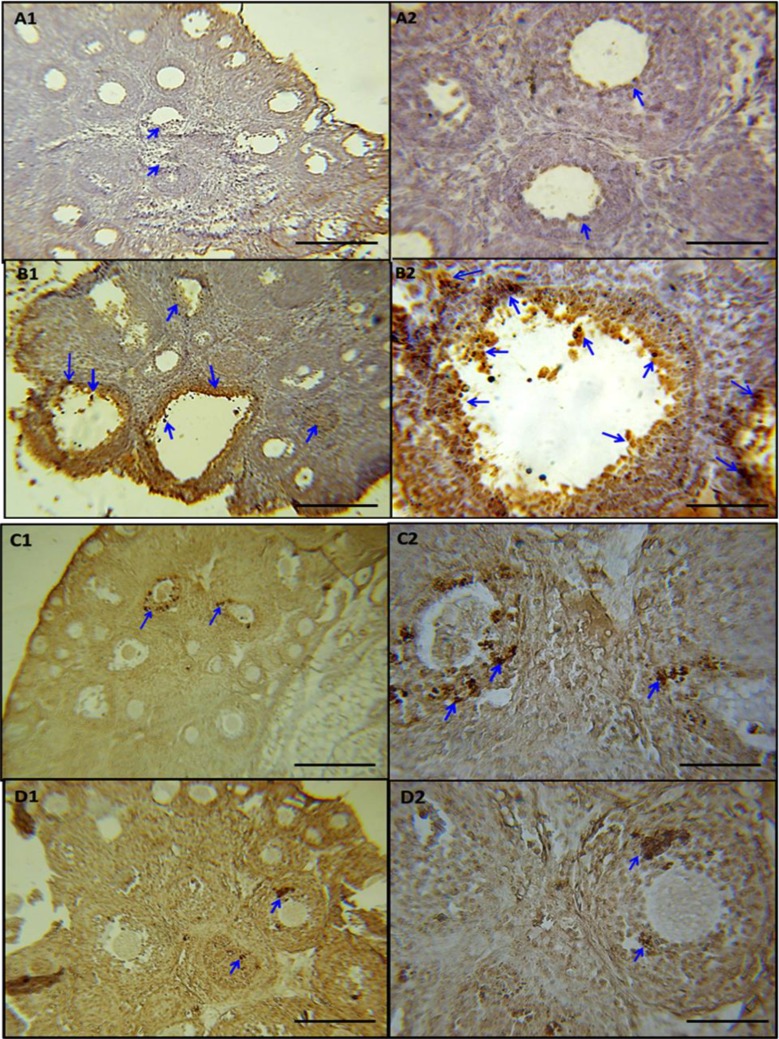
Effect of propolis on the number of TUNEL positive granulosa cells (in 0.022 mm2 of the ovary surface) in the ovaries of a rat with neonatal stress. (A1, A2) Sham group, (B1, B2) stress group, (C1, C2) stress+100 mg/kg propolis extract group and (D1, D2) stress+200 mg/kg propolis extract group. The arrows indicated by TUNEL positive granulosa cells in the ovarian tissue. TUNEL positive granulosa cells in the stressed group (B1, B2) compared to other groups increased and propolis extract (C1, C2, D1, D2) these cells is reduced. Magnification 100X and 400X, a bar in figures A1, B1, C1, and D1=250 µm and bar in figures A2, B2, C2, and D2=100 µm

The mean number of ovarian atretic follicles in stressed rats was significantly higher than the normal groups, control and under treatment with EIP. The treatment with EIP reduced the number of atretic follicles that this reduction was significantly in the treated groups with doses of 100 and 200 mg/kg in general (F=6.544, p=0.001, Table II). The number of TUNEL positive granulosa cells in the ovaries of rat pups in the normal and control groups was significantly lower than stress group. The neonatal stress increased the number of TUNEL positive granulosa cells in the ovarian follicles and the use of EIP (50, 100, and 200 mg/kg) in the animals were under stress reduced the number of these cells (F=4.456, p=0.002, Figure 3, 4).

**Effect of postnatal stress and EIP on the Lipid peroxidation and antioxidant activity**

**Figure 5:**
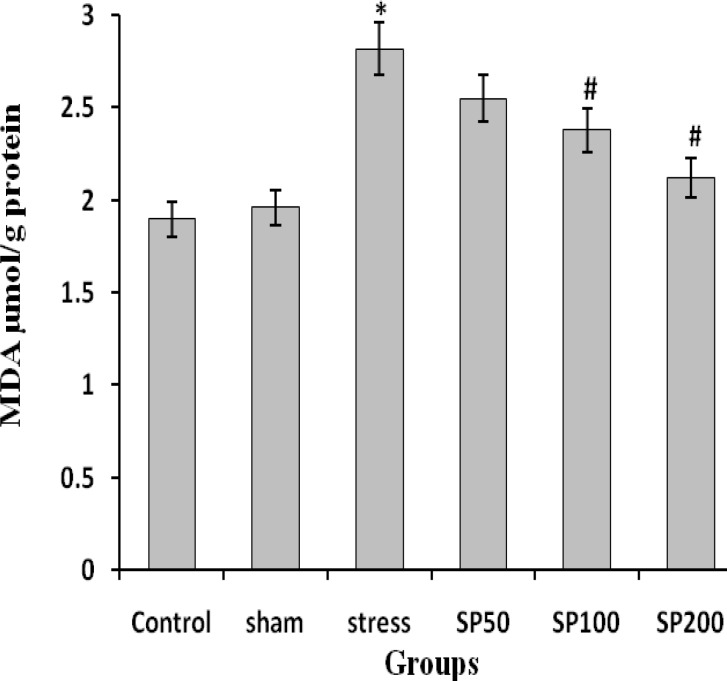
Effects of propolis on MDA (Malondialdehyde) content in ovarian tissue of rats with neonatal stress. Control group (Control), control group+saline infusion (Sham), stress group+saline infusion (Stress), stress group+50 mg/kg propolis extract (SP50), stress group+100 mg/kg propolis extract (SP100) and stress group+200 mg/kg propolis extract (SP200). * P<0.01, compared to the C and Sham groups, # P<0.05, compared to the S group, ## P<0.01, compared to the S group. All data are presented as Mean±SEM, n=8

**Figure 6:**
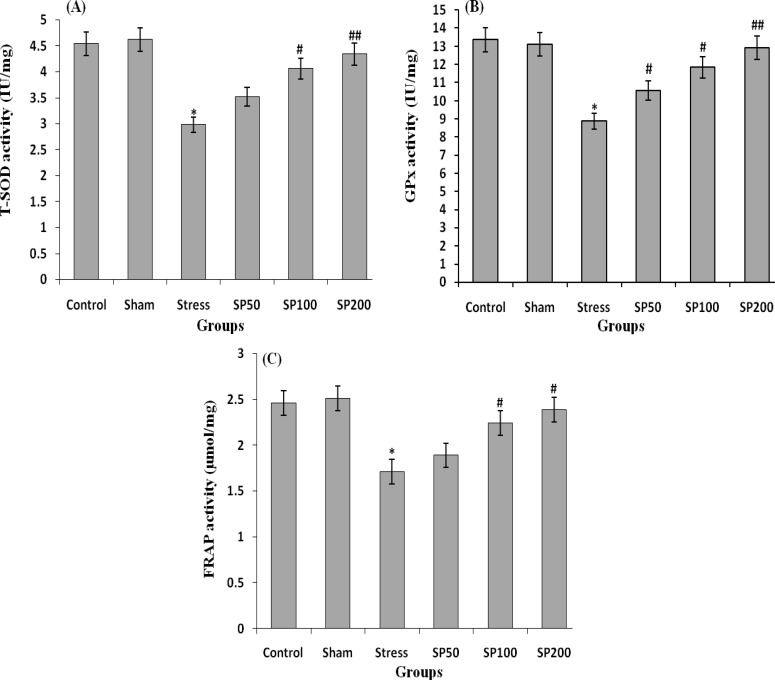
Effects of propolis on antioxidant enzyme activity in ovarian tissue of rats with neonatal stress. (A) T-SOD activity in the ovarian tissue, (B) GPx activity in the ovarian tissue, and (C) Ferric reducing antioxidant power (FRAP) in ovarian tissue. Control group (Control), control group+saline infusion (Sham), stress group+saline infusion (Stress), stress group+50 mg/kg propolis extract (SP50), stress group+100 mg/kg propolis extract (SP100) and stress group+200 mg/kg propolis extract (SP200). * P<0.01, compared to the C and Sham groups, # P<0.05, compared to the S group, ## P<0.01, compared to the S group. All data are presented as Mean±SEM, n=8

The EIP showed a strong effect on lipid peroxidation and antioxidant parameters. A significant increase in MDA and reduction in T-SOD, GPx, and FRAP concentration were observed in the stress rats as compared to the control group (F=3.324, p=0.012) (Figure 5 and 6). The increase in MDA concentration in the ovary of stress animals indicated an exasperated oxidative stress. The treatment of rats with EIP (100 and 200 mg/kg) resulted in a significant reduction in MDA and upregulation of FRAP, T-SOD, and GPx levels in the ovarian tissue compared to vehicle-treated stress rats (F=2.879, p=0.028) (Figure 5, 6).

**Table I:** Effect of postnatal stress (maternal separation stress) and Iranian propolis extract on the number and diameter of oocytes and the number of different kinds of ovarian follicles in the rat neonates

** Variables**	**Normal follicles in the ovaries (n)**				**Number of oocytes** **(at level)**	**Diameter of the oocyte (micrometer)**
**Groups**	**Primordial** **follicles**	**Primary follicle**	**Secondary** **follicle**	**Antral follicles**		
Control	119.7 ± 7.05	40.5 ± 2.96 **	11.4± 1.19 **	9.7 ± 1.32 #	153.25 ±23.63 #	22.25 ±2.6
Sham	116.6 ± 6.15	38.7 ± 5.67 **	9 ± 1.27 *	8.6 ± 1.21	164.25 ± 26.19 *	21.5 ±1.75
Stress+ Saline	67.2 ± 4.38	12 ± 1.24	3.5 ± 0.40	5.1 ± .060	71.75 ± 16.45	15.25 ± 1.6
Stress + Propolis 50	82.7 ± 4.18	31.8 ± 4.98 *	9.5± 1.1 *	8.5 ± 1.30	119.75 ±16.66	19.5 ± 2.03
Stress + Propolis 100	120.8 ± 7.52 #	33.1± 3.14 *	11 ±1.01 **	8.1 ±.085	140 ±21.32 #	24.5 ± 2.1 #
Stress + Propolis 200	124.8 ±8.97 *	42.6 ± 1.33 **	12 ±.097 **	9.9± 1.20 #	166.75 ± 25.56 *	25.25 ± 2.15 *

All data are presented as Mean ± SEM.

# : The difference between the desired group and the stress group is significant at p<0.05 level.

* : The difference between the desired group and the stress group is significant at p<0.01 level.

** : The difference between the desired group and the stress group is significant at p<0.001 level. Different as judged by Tukey–Kramer post-hoc tests.

**Table II:** Effect of postnatal stress (maternal separation stress) and Iranian propolis extract on the average number of ovarian atretic follicles in the rat neonates

** Variables**	**Atretic ovarian follicles** **(n)**		
**Categories**	**Primary follicle**	**Secondary** **follicle**	**Antral follicles**
Control	12.3± 1.12 *	8.4 ± .056 **	3.8 ± .058 **
Sham	11.25 ± 0.92 **	7.8 ±.064 **	4.2 ± .059 *
Stress +Saline	21.4± 0.86	16.6 ±.048	8.75 ± .089
Stress + Propolis 50	18.3 ± 0.67	13.25 ±.046	7.44± .047
Stress + Propolis 100	14.75 ± 0.74 #	10.8 ± .067 *	5.2± .01.05 #
Stress + Propolis 200	11.75 ±0.64 **	8.25 ±.064 **	3.4 ± .076 **

All data are presented as Mean ± SEM

# : The difference between the desired group and the stress group is significant at p<0.05 level.

* : The difference between the desired group and the stress group is significant at p<0.01 level.

**: The difference between the desired group and the stress group is significant at p<0.001 level. Different as judged by Tukey–Kramer post-hoc tests.

## Discussion

In this study, we showed that the maternal separation stress increased serum corticosterone level and decreased 17-beta-estradiol level and EIP prevented these changes. Moreover, the maternal separation stress reduced the number of primordial, primary and secondary follicles, the number, and diameter of the oocytes and treatment of stressed pups with EIP (100 & 200 mg/kg) significantly increased the number of ovarian follicles, number, and diameter of the oocytes. Our findings also showed that neonatal stress increases the number of atretic follicles and apoptotic granulosa cells (TUNEL positive) by intensifying atresia process and the treatment of the pups with EIP reduces the number of atretic follicles and apoptotic granulosa cells by decreasing the intensity of follicular atresia process.

Increased serum corticosterone level in young rats due to the separation from the mother represents one of the important conditions of stress (31). In addition, loss of body weight is expected in stressed young rats as stress inhibits feeding behavior. The treatment of the stressed neonates with EIP at all used doses prevented the body weight loss of the animals. These protective effects of propolis were associated with the decrease of corticosterone level and an increase of the of the 17-beta-estradiol level. Previous findings have shown that the stress reduces gonadotropins which are as an apoptotic inhibitor factor in granulosa cells (9).

Thus, the apoptosis induction by stress and subsequently the reduction in the number of granulosa cells reduces the biosynthesis of 17-beta-estradiol and creates hypo-estrogenic conditions in the ovary (32). The reduction of 17-beta-estradiol level decreases the ovum quality through its apoptosis induction. Our hypothesis is that the neonatal chronic stress induces the apoptosis of granulosa cells and may reduce 17-beta estradiol biosynthesis, and consequently reduce the quality of the ovum. Our results are in agreement with the findings reported by Okamoto *et al*, showed that oral administration of propolis induces estrogenic activity in estrogen target organs in vivo suggesting that propolis is a useful dietary source of phytoestrogens and a promising treatment for post-menopausal symptoms (33).

Propolis is rich in antioxidants such as polyphones and flavonoids (13-34). The composition of propolis depends upon the vegetation of the area from where it was collected. Colorimetric methods are convenient and appropriate for the routine analysis of phenolics (35). Antioxidant activity has also been demonstrated in propolis. It’s proposed that strong antioxidant activity occurs in propolis with high amounts of phenolic compounds and weak activity happens with low amounts (36). Total polyphone content of ethanol extracts of this sample was (6.4 gr/100g±0.03). According to the other studies, total polyphone content of this extract is more of them.

The several reports have shown that the ovaries of the female rats contain only the oocytes immediately after birth and are without any follicles, and this means that follicular development and evolution process occurs after the birth in the rats (29, 33). As well, it is known that primordial follicles are created three days after the birth and lead to the formation of primary follicles immediately after their growth and differentiation process (32). Our studies showed that stress causes reduction in the different kinds of ovarian follicles including, primordial, primary and secondary follicles that the findings reported by Bhatt and *et al* confirm it (19, 20). On the other hand, the maternal separation stress significantly increased the number of atretic follicles in the ovaries of the neonates and this is along with previous reports (37, 38). It seems that the reduction of ovarian follicles and increase of atretic follicles are probably resulting from the impaired secretion of gonadotropins and steroids (17-beta-estradiol) followed by oxidative stress induced by the chronic stress conditions.

In the present study, propolis, as one of the most powerful natural antioxidants (its antioxidant power is four times vitamin E), is likely able to prevent the destruction and the follicular atresia and ultimately protects the reduced number of ovarian follicles. Previous findings have shown that when vitamins E and C are used as antioxidants to cure the animals treated with Methidathion (an agent of lipid peroxidation in the ovary and an agent of increasing atretic follicles), they significantly reduce the number of atretic follicles (39).

The chronic stress reduces the production and secretion of gonadotropins FSH and LH that are necessary for vital, growth and development of ovarian follicles and oocytes by stimulating the activity of hypothalamus-pituitary-adrenal axis (40). The effect of stress on the adult gonads may be irreversible in the early stages of follicular development (pre-puberty). The increase secretion of ACTH inhibits follicular development and ovulation (40). So, reducing the amount ACTH disrupts follicular development process and oocyte maturation, which eventually alters the number and size of oocytes.

Stress as one of the factors that drastic changes in the level of glucocorticoid (corticosterone in rodents) and steroids hormones are created and played an important role in increasing the rate of follicular atresia. The formation of free radicals increases by raising the levels of glucocorticoids in the stress state and consequently, the production of sex steroids is reduced. ROS may play an important role in the survival and development of ovarian follicles. Therefore, their increases may cause drastic reduction in primordial follicles in the ovaries. ROS may play an important role in beginning the apoptosis process of follicles, including antral follicles. As well reducing the amount of glutathione (as a cellular antioxidant) causes to stimulate follicular atresia and apoptosis in granulosa cells of the follicles. However, previous studies have shown that estrogen has antioxidant properties, and therefore its deficit during stress may lead to the production of ROS (41-44). In this study, using EIP caused to decrease ROS production and subsequently to reduce the apoptosis of granulosa cells and follicular atresia, possibly by reducing the levels of corticosteroids and increasing the production of ovarian steroids.

Propolis is one of the richest sources of plant phenols (flavonoids and phenolic acids), which are widely recognized as powerful antioxidants (12, 19, 44-47). Various flavonoids and phenolic are able to take the free radicals and protect the lipids and other compounds such as vitamin C against Oxidation or the destruction during oxidative stress (9, 45). It has been reported that the enzymatic and non-enzymatic activities of antioxidants have been significantly increased using propolis; as well propolis has induced a significant increase in the level of vitamin C in plasma, kidney, stomach, small intestine, and colon (48). It has been proven that propolis is absorbed from the blood circulation and plays a role as a hydrophilic antioxidant to absorb vitamin C (48). In addition to flavonoids, a compound called caffeic acid phenethyl ester (CAPE) is also responsible for the antioxidant properties of propolis; it was reported this compound protects the cell membrane against lipid peroxidation. Previous studies have shown that CAPE reduces malondialdehyde (MDA) level by suppressing the production of an oxygen free radical as an antioxidant (49).

To further investigate the mechanism of action of propolis, we measured the anti-oxidative capacity and the activity of FRAP. We examined the total antioxidant activity in the ovary tissue using FRAP as an indicator of the strength of non-enzymatic antioxidants. The results showed that in a postnatal stress rat ovary, there is a significant decrease in the antioxidant activity (FRAP) in stress group and treatment with the EPI (100 and 200 mg/kg) enhanced the antioxidant capacity of the FRAP. SOD neutralizes the superoxide anions by converting them into hydrogen peroxide and GPx reduces hydrogen peroxide to water; together SOD and GPx serve as an antioxidant defense mechanism (47, 48).

## Conclusion

These findings suggest that propolis may prevent the destructive effects of psychological stress (maternal separation of pups) as well as reduce the structural and developmental changes in the ovaries of rat neonates using its strong antioxidant effects due to the compounds such as flavonoids and polyphenols. Thus, propolis with more powerful antioxidant properties might be used as a compound with therapeutic potential protection against damage caused by stress.

## Note

***This article extracted from M.Sc. thesis. (Atefeh Arabameri)***
